# Molecular Defects in Friedreich’s Ataxia: Convergence of Oxidative Stress and Cytoskeletal Abnormalities

**DOI:** 10.3389/fmolb.2020.569293

**Published:** 2020-11-09

**Authors:** Frances M. Smith, Daniel J. Kosman

**Affiliations:** Department of Biochemistry, State University of New York at Buffalo, Buffalo, NY, United States

**Keywords:** Friedreich’s ataxia, frataxin, Nrf2, PIP5K1β, cytoskeleton, glutathione

## Abstract

Friedreich’s ataxia (FRDA) is a multi-faceted disease characterized by progressive sensory–motor loss, neurodegeneration, brain iron accumulation, and eventual death by hypertrophic cardiomyopathy. FRDA follows loss of frataxin (FXN), a mitochondrial chaperone protein required for incorporation of iron into iron–sulfur cluster and heme precursors. After the discovery of the molecular basis of FRDA in 1996, over two decades of research have been dedicated to understanding the temporal manifestations of disease both at the whole body and molecular level. Early research indicated strong cellular iron dysregulation in both human and yeast models followed by onset of oxidative stress. Since then, the pathophysiology due to dysregulation of intracellular iron chaperoning has become central in FRDA relative to antioxidant defense and run-down in energy metabolism. At the same time, limited consideration has been given to changes in cytoskeletal organization, which was one of the first molecular defects noted. These alterations include both post-translational oxidative glutathionylation of actin monomers and differential DNA processing of a cytoskeletal regulator PIP5K1β. Currently unknown in respect to FRDA but well understood in the context of FXN-deficient cell physiology is the resulting impact on the cytoskeleton; this disassembly of actin filaments has a particularly profound effect on cell–cell junctions characteristic of barrier cells. With respect to a neurodegenerative disorder such as FRDA, this cytoskeletal and tight junction breakdown in the brain microvascular endothelial cells of the blood–brain barrier is likely a component of disease etiology. This review serves to outline a brief history of this research and hones in on pathway dysregulation downstream of iron-related pathology in FRDA related to actin dynamics. The review presented here was not written with the intent of being exhaustive, but to instead urge the reader to consider the essentiality of the cytoskeleton and appreciate the limited knowledge on FRDA-related cytoskeletal dysfunction as a result of oxidative stress. The review examines previous hypotheses of neurodegeneration with brain iron accumulation (NBIA) in FRDA with a specific biochemical focus.

## Prologue

This review re-examines the under-appreciated molecular manifestation of Friedreich’s ataxia (FRDA) associated with oxidative modification of the cytoskeleton. As the foundation of the cell body, facilitator of intracellular signaling cascades, anchorage of junctional proteins, and driving force for motility and contractility, proper actin dynamics are essential to maintaining cell homeostasis. Oxidative stress is likely an aspect of the cellular manifestations of FRDA and early reports demonstrated cytoskeletal abnormalities correlated with increased glutathionylation of actin. However, the downstream effects of this modification are not well documented and the consequences of this cytoskeletal disruption have not been elucidated. Thus, actin dysregulation in FRDA is known as a pathophysiological consequence of disease, but knowledge of the cellular implications of this phenotype is lacking.

We first provide the background essential to our examination of the role of dysregulation of the cytoskeleton in the cellular pathology associated with FRDA. Thus, this review addresses first the abnormal iron accumulation characteristic of FRDA, specifically the iron accumulation in the heart and central nervous system (CNS). The molecular abnormalities in the cell due to this impaired iron metabolism will be reviewed and how these lead to accumulation of the pro-oxidant ferrous iron. The resulting redox stress in FRDA contributes to the following abnormalities: (1) aberrant Nrf2 signaling in antioxidant production; (2) increased ferroptotic susceptibility; and (3) increased protein—actin—glutathionylation, the focus of this review. Although reversible, glutathionylation becomes pathologic during prolonged oxidative stress and severely impairs actin’s polymerization capacity and the affinity for tropomyosin essential to the stability of actin filaments (F-actin). Although FRDA research has examined actin dynamics sparingly, we present the current understanding of this aberrant actin metabolism, in particular, the loss of polymerization capacity on the one hand, and of PIP5K1ββ, a phosphoinositol kinase essential to filament assembly. The objective of this review is to highlight the lack in knowledge of actin dysregulation in FRDA in the context of the wealth of information about its molecular origins and pathology. While documented nearly two decades ago, how actin glutathionylation in response to the cell redox stress that is part of this pathology remains a key unknown in the etiology of this genetic disorder. We have compiled compelling research to reinforce the necessity of detailing the cerebellar dentate nucleus (CDN) iron accumulation co-morbid with neurodegeneration in FRDA.

## Introduction

### The Molecular Basis of FRDA: The Essentiality of Frataxin

**FIGURE 1:**
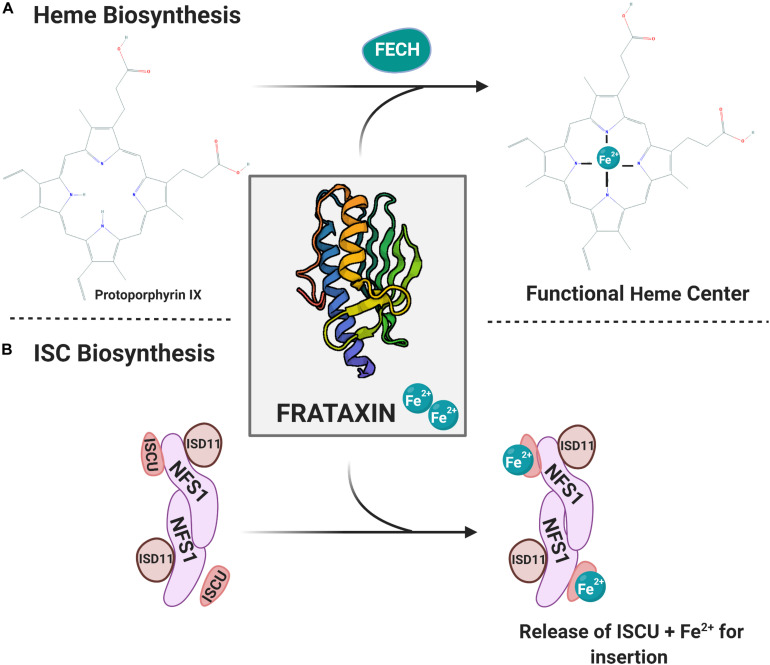
Mitochondrial iron co-factor biosynthetic pathways are reliant on FXN iron chaperone function. **(A)** Heme biosynthesis. The final step is ferrochelatase (FECH)-mediated Fe^2+^ insertion into protoporphyrin IX. FXN-Fe^2+^ serves as the iron source for this heme maturation process. **(B)** The mitochondrial iron–sulfur cluster (ISC) biosynthetic pathway is depicted. The ISC scaffold includes NFS1, ISD11, and ISCU dimers. Delivery of the Fe^2+^ by FXN follows sulfur addition by cysteine desulferase (not shown). The final step of the formation before release of ISCU containing ISC centers for addition into an apo-enzyme. Protein structure provided through The Protein Data Bank (Berman et al., 2000; Dhe-Paganon et al., 2000).

FRDA arises from a failure of transcription and post-transcriptional processing of the frataxin (FXN) gene. Human FXN is a nuclearly encoded protein of 210 amino acids that is processed by the mitochondrial peptidase-β to its mature form of 120 residues (Campuzano et al., 1996; Musco et al., 2000). Its function is specific to the inner mitochondrial compartment where it acts as an iron chaperone protein, facilitating assembly of iron–sulfur clusters (ISC) and the metalation of protoporphyrin IX in the biosynthesis of heme (Rötig et al., 1997; Park et al., 2003; Bulteau et al., 2004; Yoon and Cowan, 2004; Cai et al., 2018). FXN interacts with ISD11 in the ISC biosynthetic pathway and with ferrochelatase in heme synthesis as illustrated in Figure 1 (Yoon and Cowan, 2004; Shan et al., 2007). The ISC is one of the evolutionarily oldest prosthetic groups most commonly facilitating an electron transfer process in support of a complex redox biology. Among other things, ISCs are essential to energy metabolism in the electron transport chain (ETC). FXN’s localization to the mitochondria and its function in providing enzymatic function to energy metabolism pathways highlights its essential role. In addition, FXN has both physical and functional interactions with ETC complex II, facilitating respiration (Gonzalez-Cabo et al., 2005). FXN has also been implicated as a mitochondrial iron storage protein in iron overload based on its exposed β-sheet (Yoon and Cowan, 2003; Bencze et al., 2006). The β-sheet hypothesis suggested that this interface interacts in electron transfer during ISCU scaffold protein interaction for ISC formation. FXN is ubiquitously expressed throughout all tissues of the body and is positively correlated to tissues with high requirements of energy metabolism. Babcock et al. (1997) noted that post-mitotic, heavily aerobic cardiac muscle cells and neurons are heavily targeted in human disease. Thus, FRDA is considered a disease of both altered iron metabolism and respiratory deficiency. However, these phenotypes alone do not explain why other highly aerobic tissues and motor neuron types are spared in disease. FXN is most heavily studied in cardiac skeletal muscle, neurons, and fibroblasts for *in vitro* examination of cell disease profiles. Being strongly affected in FRDA, the former two cell types prioritizes their study—although acquisition of these cells from patients are invasive (cardiac tissue) or inaccessible (neurons).

### Yeast Frataxin Homolog (YFH1)

FXN is highly conserved and found as early as in photosynthetic purple bacteria (CyaY); the human and the yeast homolog, YFH1, exhibit 65% sequence similarity (Gibson et al., 1996; Bencze et al., 2006). In yeast, disruption of the *YFH1* gene prevents growth on glycerol and ethanol non-fermentable carbon-source media in comparison with proper growth on fermentable glucose media (Wilson and Roof, 1997). This was one of the first demonstrations that a lack in FXN machinery creates a disease of respiratory function. Further examination indicated respiratory function fourfold decreased in the mutants than the WT and a complete lack of mitochondrial DNA (mtDNA) (Wilson and Roof, 1997). Multiple components of the mitochondrial respiratory complexes are encoded by mtDNA, indicating the necessity of mtDNA for respiratory function. These preliminary studies of YFH1 served as the foundation for research of the necessity of FXN in higher eukaryotes.

Babcock et al. (1997) published an examination of YFH1 mutant homeostatic profiles in response to multiple exogenous stressors, which again showed growth only on fermentable media, temperature sensitivity, and reduced oxygen consumption. The YFH1 mutants show striking growth parallels to *Rho*- yeast, which have only partial fragments of mtDNA and co-incident defects in oxidative phosphorylation (oxphos). Concerning iron metabolism, the ΔYFH1 mutants had an increase in both low- and high-affinity iron uptake mechanisms and 10-fold increased mitochondrial iron content. In combination with a halved rate of cell division, this indicates that defects in FXN/YFH1 alters iron metabolism and/or intracellular accumulation. To further reinforce that YFH1/FXN is essential in iron metabolism and mitochondrial function in eukaryotic cells, YFH1 overexpression rescues a growth defect of *bm-8* mutant yeast cells which are unable to grow on iron-deplete media (Babcock et al., 1997). Toxicity of the iron accumulation in ΔYFH1 cells was exacerbated by treatment of hydrogen peroxide, indicating sensitivity to oxidative stress with a lack of YFH1 (Babcock et al., 1997). Early yeast studies also indicated functional interaction of YFH1 with proteins of ETC complexes as well as electron transfer flavoprotein ETF-α and -β; this was corroborated with human equivalents of FXN interacting with complex II subunits A and B (Babcock et al., 1997; Gonzalez-Cabo et al., 2005). Nonetheless, yeast studies first defined the biochemical foundation of FRDA pathology.

## The Foundation of FRDA Pathology

FRDA was first described in 1863 by Nikolaus Friedreich, a pathologist and neurologist who described a disease with loss of linguistic ability, coordination, sensory–motor innervation, peculiarly slow disease progression, early age of onset, and familial inheritance. Consistent with pathological manifestations, Friedreich noted in postmortem examinations the degeneration of the dorsal root ganglion (DRG), demyelination in multiple sections of the brain, and extensive atrophy to the heart (Pearce, 2004). Later understanding of FRDA includes changes in pancreatic function, leading to the development of diabetes in about 10% of patients and glucose intolerance in 20% (Melo et al., 2005). FRDA has a mean age of onset at 10–15 years with a mean age of death at 37 (Dürr et al., 1996).

MRI of patients throughout disease progression has revealed significant differences in patient CDN compared with healthy controls, including increased iron accumulation and gross tissue atrophy (Ward et al., 2019). Iron deposits are also commonly seen in the heart accompanying fibrosis and the onset of cardiomyopathy. These whole-body manifestations supported the hypothesis that altered iron homeostasis was central to FRDA pathology (Sanchez-Casis et al., 1976; Lamarche et al., 1980; Ramirez et al., 2012). The dyshomeostasis in cellular iron metabolism in FRDA renders this disease a case of both regional iron deficiency and overload, as will be discussed throughout this review; mitochondrial iron accumulation is at the expense of the cytosolic iron pool (Babcock et al., 1997; Wilson et al., 1998). Increased levels of circulating transferrin receptor (TfR) found in FRDA patient blood plasma levels reflect depletion of intracellular iron (Wilson et al., 2000). FRDA patients exhibit decreased blood plasma-iron levels and cytosolic ferritin levels shifted to higher concentration of its light-chain than heavy-chain counterparts in the dentate nucleus, indicating iron transport from the circulation but bypass of cytosolic storage (Koeppen et al., 2007; Pathak et al., 2019). Furthermore, FRDA patient heart, liver, and spleen sections analyzed for iron content using Perl’s stain demonstrate extra-nuclear iron deposition, and in a chain-like organization, indicating mitochondrial localization (Bradley et al., 2000).

Similarly, a FRDA mouse model of cardiac and skeletal muscle FXN loss demonstrates in cardiac tissue: increased TfR, decreased cytoplasmic ferritin heavy and light chain subunits, increased mitochondrial iron importer mitoferrin-2 (mfrn-2), and decreased expression of the iron exporter ferroportin (FPN) (Huang et al., 2009). These data on altered iron trafficking, though only in mouse models, represent an iron deficiency in the extracellular environment and in the cytoplasm with an increased transport to the mitochondria. Challenges arise in using mouse models for FRDA because intron 1 of mouse FXN does not contain GAA expansion tracts, and thus mice do not naturally develop this disease. In addition, complete FXN-knockout mice are embryonic lethal, and thus our best mouse models are those which are temporally conditional FXN deficient or those which exhibit tissue-specific FXN loss (Cossée et al., 2000). Mouse models lack consistency to human disease by exhibiting increased transferrin-bound iron in the serum, which in the model of cardiac and skeletal muscle FXN-deficient mice may reflect physiological iron concentrations and function of all other organ and tissue systems (Whitnall et al., 2012). This is of course in contrast to FRDA patients which experience global and progressive FXN knockdown.

Mfrn-2 upregulation is also seen in *Drosophila melanogaster* models of FXN deficiency, and modulation of its levels is positively correlated to reversal of both iron dysregulation and disease severity (Navarro et al., 2015). It remains unknown if mfrn-2 levels are altered in FRDA patients, although skeletal muscle biopsies of ISCU myopathy patients and siRNA-ISCU fibroblasts transcriptionally upregulate mfrn2 (Crooks et al., 2014). Mfrn-2’s involvement in mitochondrial iron homeostasis may explain the increased iron trafficking from the extracellular environment and cytoplasm into the inner mitochondrial compartment seen in FRDA. Collectively, these observations indicate increased cellular iron import, by-pass of cytosolic storage, and increased mitochondrial import (Babcock et al., 1997; Pandolfo and Hausmann, 2013). Thus, iron is of central importance in FRDA and is a focus of this review.

### The Whole-Body Manifestations of FRDA

#### The Genetic Basis of FRDA

A wealth of primary research on FRDA began after the discovery of the molecular basis in 1996. Campuzano et al. (1996) identified the dysregulation in frataxin (FXN) protein due to the accumulation of GAA repeat expansions localized to the first intron of the FXN locus in patients. FXN and the other biomolecules key to FRDA and referred to in this review are listed in Table 1. The FXN gene typically contains 8–34 GAA triplets; pathological onset is linked to 40–60 repeats that can increase to over 1700 (Dürr et al., 1996; Cossée et al., 1997). It is now understood that the number of repeats is positively correlated with earlier onset and disease severity, which worsens with time due to continual cell replication and diminishment of the FXN pool (Dürr et al., 1996). The expansions are a hallmark of disease as most patients are homozygous for this mutation; others are compound heterozygous with the expansion and a point mutation, exonic deletion, or whole allelic deletion in the other allele (Anheim et al., 2012; van den Ouweland et al., 2012). Several point mutations as well as missense mutations result in lack of protein production or processing (Li et al., 2013; Saccà et al., 2013; Heidari et al., 2014; Clark et al., 2017). In addition, repressive chromatin marks indicate a role for epigenetic FXN silencing in disease (Herman et al., 2006).

**TABLE 1:** Proteins and metabolites key to the pathology of FRDA.

Name	Gene symbol	Function	FRDA relevance
Aconitase	ACO2	Citrate-isocitrate conversion in TCA cycle (mitochondrial), modulates IRE-containing gene transcription (cytoplasmic)	ISC containing, decreased activity in FXN deficiency
Cysteamine/Cystamine		Provides cysteine in glutathione synthesis	Anti-oxidant, potential rescue of glutathione metabolism in FXN deficiency
F-actin	ACTB	Cytoskeleton formation, anchorage of junction proteins, anchorage of organelles	Lack of polymerization and tropomyosin binding in FXN deficiency. Gives cells altered morphology
Frataxin	FXN	Iron incorporation into ISCs and heme centers	Decreased transcription and protein production in FRDA Inefficient ISC and heme biosynthesis
Glutathione Effectors: Glutaredoxin (Grx) Glutathione-S-Transferase (GST) Sulfiredoxin (Sfx) Thioredoxin (Trx) Peroxiredoxin (Prx)	GLRX GSTP/M/A/T SRXN1 TXN PRDX1/2/6	GSSG → (2)GSH GSH + HSP → GSSP S-S → (2)SH S-S → (2)SH S-S → (2)SH	FXN-deficiency promotes increased glutathionylation of proteins. Cell GSH buffering capacity depressed due to pathologic oxidative stress. Actin glutathionylation disrupts protein-protein interactions in actin polymerization and anchorage of tight junction proteins
ISD11	LYRM4	ISC scaffolding protein, functional interaction with FXN	A lack of FXN prevents efficient ISC cluster formation at the step of iron insertion
Keap1	KEAP1	Tethers Nrf2 proteins to actin bundles, preventing ARE-containing gene transcription	Loss of Keap1 binding to actin filaments in FRDA prevents tethering of Nrf2, preventing its activity as a transcription factor
Nrf2	NFE2L2	Oxidative stress-activated transcription factor for ARE-containing genes	Decreased activation of downstream antioxidant and iron metabolism proteins with FXN deficiency
PIP5K1β	PIP5K1B	Actin stabilization protein	Less PIP5K1 β protein production in FRDA due to *cis*-silencing, destabilizes actin filaments by loss of PI(4,5)P_2_
Protoporphyrin IX	–	Final metal-free precursor of heme	Possible accumulation in absence of FXN Fe-delivery
ZO-1	TJP1	Scaffold protein of TJs which tethers peripheral junctional proteins to actin	Loss of junctional continuity at the cell periphery in models of mitochondrial stress and depressed oxidative phosphorylation

*A table of the key biomolecules highlighted in this review which contribute to the pathology of FRDA, as well as relevant proposed drug candidates. Gene symbol, function, and dysregulation in FRDA pathology are included where appropriate. See text for argumentation of FRDA and FECH interactions during heme biosynthesis. This table was designed with the intention of adequately reflecting the important focal points described in this review.*

FRDA mostly effects the Caucasian population with an incidence of about 1:50,000 and is inherited in an autosomal recessive manner; 1:60–1:120 individuals are thought to be a carrier of the underlying mutation (Santos et al., 2010). FXN is a ubiquitously expressed protein, thus having the potential to affect all tissues. One may then question the tissue specificity in disease, largely targeted to cardiac and central nervous system tissues, which is currently attributed to somatic instability. With successive rounds of DNA replication in mitotic and highly metabolic cells, one could expect that the heart is largely affected, consistent with patient death. Indeed, analysis of patient tissues for GAA expansion length from the heart, cerebral cortex, spinal cord, cerebellar cortex, and pancreas show the strongest expansion in the heart and pancreas, two tissues heavily implicated in FRDA pathology (Long et al., 2017). The post-mitotic nature of neurons may explain the lack of significant GAA expansion but paradoxical susceptibility in disease.

The GAA expansion starts early at a non-pathological level, but accumulates during successive rounds of DNA replication due to nucleotide slippage, creating secondary structures which hinder processing during transcription (Heidenfelder et al., 2003; Li et al., 2015; Long et al., 2017; Khristich et al., 2020). Net contractions may result from DNA excision mechanisms while continual expansion occurs upon transcription. Thus, FRDA is largely a disease of decreased expression levels of FXN in the expansion model, and loss-of-function of FXN in the non-expansion mutations discussed. This compromises the function of the FXN protein as an iron chaperone, with severe metabolic consequences, as is discussed throughout this review. Other repeat expansion disorders include Huntington’s disease (HD) and Fragile X syndrome (FXS), all of which are associated with forms of neurodegeneration (FRDA and HD) or mental retardation (FXS) consistent with atrophied areas of brain tissue (Brunberg et al., 2002; Kassubek et al., 2004; Ward et al., 2019). This highlights the sensitivity of the CNS to diseases with such profound protein dyshomeostasis.

#### Cardiomyopathy in FRDA

Cardiomyopathy is the most common cause of death in FRDA patients (∼59%) and typically occurs after two decades of disease progression (Tsou et al., 2011). Cardiomyopathy includes fibrosis of cardiac wall tissue at later disease stages with thickening of the left ventricle wall (Hanson et al., 2019). Patients often succumb to disease due to resulting arrhythmia and dilated cardiomyopathy (Casazza and Morpurgo, 1996; Kipps et al., 2009). Cardiomyocytes have been identified to be deficient in cell oxphos secondary to a lack of FXN; in addition, cardiomyocyte mitochondria have heightened iron deposits (Sanchez-Casis et al., 1976; Lamarche et al., 1980; Rötig et al., 1997; Ramirez et al., 2012). The presence of iron accumulation in multiple tissues including the heart and brain raises the question of why particular organ systems are targeted in disease. Cardiomyocytes have high and sustained energy demand and thus may more readily become energy starved due to a loss of functioning FXN. Iron accumulation in tissues of high energy demand is a common aspect of FRDA, as will be further demonstrated in the following section that reviews the CNS iron accumulation. Currently, most of the literature on the heart pathology linked to FXN focuses on the key role FXN plays in iron metabolism in cardiomyocytes, with little consideration of FXN function in cardiac vascular endothelial cells, for example. While the vascular endothelial cells may or may not have a contributory role in the onset of cardiac pathology, proper function of endothelial cells is indispensable for selective access of blood solutes to vital organs including the heart. These cell types are the focus of this review.

#### Neurological Defects in FRDA

The iron accumulation in the CDN was noted earlier, but the corresponding impacts to pathophysiology remain unclear. Thus, precise molecular defects underlying the degeneration of the CDN and the peculiar hyperintensity due to iron remain an open question with respect to the neuropathology in FRDA. In addition, due to the inherent low resolution of MRI in terms of depicting cellular let alone sub-organellar distribution of metal, the exact localization of the iron deposits, even intracellular vs. extracellular, remains unknown. However, the increased iron concentration in the CDN imaged by MRI remains today as one of the key diagnostic criteria for diseases presenting as neurodegeneration with brain iron accumulation (NBIA) (Schipper, 2012). FRDA is often loosely classified but not strictly associated as an NBIA disorder as it lacks the classical deposition in the basal ganglia and infrequently is linked to extrapyramidal movement dysfunction (Kruer and Boddaert, 2012). Unsurprisingly, two other proteins involved in iron homeostasis, ceruloplasmin (ferroxidase activity) and ferritin (iron storage and trafficking), are linked to two classical NBIA diseases, aceruloplasminemia and neuroferritonopathy, respectively (Hogarth, 2015). In FRDA patients, the DRG and CDN are strongly atrophied essential areas of high-velocity cognitive processing. The DRG is important in perceiving pain innervation and is responsible for coordinating peripheral and CNS signaling. The CDN is essential in coordination of sensory–motor innervation and is responsible for fine motor movement and corrected movement. This high-velocity cross-talk represents a large neuronal energy demand (Habas, 2010; Haberberger et al., 2019). This becomes pathological for tissues which are highly metabolic and therefore strongly dependent on the function of FXN.

Waldvogel et al. (1999) used MRI to quantify brain iron concentration in the CDN and pallidum. The pallidum are of interest as they are the most iron-rich structures of the human brain. At the time of this report, it was understood that brain iron accumulates naturally with age in humans, and mostly within the first 30 years of life. Thus, this paper sought to examine the presence of and rate of iron changes in FRDA patient brains relative to controls (Waldvogel et al., 1999). FRDA patient CDNs showed elevated iron loading and a greater gradient of iron accumulation as a function of age whereas the pallidum showed no significant differences in iron concentration at all. Furthermore, the differences in iron concentration between FRDA patients and controls seemed to be more significant in the younger patients, correlating disease severity to early disease onset. Although this paper did not pinpoint the exact role of iron in CDN pathology of FRDA, it reinforced the centrality of iron to CDN disease manifestation. Interestingly, it suggested a dichotomy with respect to iron sensitivity in the CNS because brain iron concentration in the iron-rich pallidum accumulated over time as expected, but with no significant differences from the control brains, as is normal of aging (Waldvogel et al., 1999). It is also worth mentioning that other iron-rich structures of the brain, including the substantia nigra, red nucleus, and subthalamic nucleus, are spared in disease (Koeppen et al., 2007). Developments in research essential to understanding these phenomena include sensitive detection of (1) sub-cellular localization of magnetic species, (2) intra- or extracellular locale, or (3) distinguishing between soluble, ferrous iron, or insoluble iron bound to ferritin or hemosiderin.

In 2007, Koeppen et al. (2007) argued that FRDA CDN iron is more reorganized among inner-brain cell types. By gross examination of FRDA and control postmortem brains using Perl’s Prussian blue stain which detects labile iron, Koeppen et al. (2007) saw less and more diffuse iron staining in FRDA brains. Biochemical quantification of ferritin as an indirect method of quantifying brain iron showed a shift in the amount of ferritin light chain (the storage part of the protein) relative to ferritin heavy chain in FRDA brains. Thus, discrepancies in detection methods challenge the true understanding of brain iron in FRDA. Furthermore, the immunofluorescence detection of ferritin showed a strong co-localization with astrocyte and microglial markers, supporting that brain iron is reorganized in FRDA (Koeppen et al., 2007). This is accompanied by increased presence of microglia in FRDA brains; these cells are specialized CNS-specific phagocytosing cells which remove toxins, debris, and remnants of cell death. Their increased presence may presumably reflect the increased need to remove dead, FXN-deficient neurons. Interestingly, regions of the neuropil, unmyelinated neuronal networks, had collapsed but were highly reactive for ferroportin immunostaining. Again, this was indicative of a reorganization of brain iron with heightened staining in FRDA brains relative to controls (Koeppen et al., 2007). Notably, this paper analyzes the endpoint of disease, further compounded by promotion of iron–ferritin dissociation during the lag time between death and tissue fixation. Conversely, MRI analysis better interrogates iron’s centrality in the pathology of disease progression. Importantly, iron quantification by MRI measures that bound to both ferritin and hemosiderin, which Perl’s staining misses. The combination of MRI for tracking iron accumulation over time, and Perl’s staining for quantitation after death and tissue analysis would have clear advantages for assessing the comorbidity of iron accumulation in FRDA.

Bonilha da Silva et al. (2014) longitudinal study using T2 MRI to compare iron concentrations in the CDN, substantia nigra, putamen, cerebellar white matter, and caudate nucleus between FRDA patients and controls. Significant increases in iron loading only occurred in the CDN of FRDA patients compared with controls, and the iron content was positively correlated to GAA expansion number and the FARS rating score, which is used as a scoring spectrum for disease severity in FRDA patients. This study laid the groundwork for the importance of longitudinal examination of CDN iron content in FRDA patients and its use in diagnostic criteria (Bonilha da Silva et al., 2014).

Ward et al. (2019) longitudinal study imaged separately CDN atrophy and iron accumulation throughout FRDA disease progression *via* quantitative susceptibility mapping (QSM)-MRI in a 2-year study. By separately examining the iron content and atrophy in the CDN in a temporally linear manner, different stages of disease can more easily be assessed. QSM provides more sensitive detection of metal-rich tissues and is therefore a desirable neuroimaging technique. QSM-MRI was used to examine the following aspects sensitively and temporally through disease finding: (1) changes in iron concentration and (2) tissue volume of the CDN as a function of tissue atrophy (Ward et al., 2019). Tissue volume is essential in neuroimaging as it can influence metal detection—the same amount of iron particles in a smaller amount of tissue gives a higher reading than in a larger tissue volume.

Ward et al. (2019) found that brain atrophy in FRDA begins early in disease and plateaus in later stages, indicating that the neurodegenerative profile is an early-onset disease manifestation. In contrast, the iron accumulation was progressive throughout the study and continued after loss of the large dopaminergic neurons, suggesting that the iron accumulation persists even after the dentate loses tissue volume. Furthermore, the rate of BIA increased throughout disease progression. Both atrophy and iron accumulation correlated with the baseline FARS score of patients. Thus, Ward et al. (2019) concluded that iron dysregulation in the CDN of FRDA patients continued throughout disease progression independent of the early onset of neuronal degeneration. Importantly, this study challenged the previous redistribution hypothesis of brain iron (Koeppen et al., 2007). Significantly, this suggests that iron deposition in the CDN of FRDA patients is a progressive etiology of the disease.

### Neuronal Sensitivity

The natural enrichment of iron in the CDN may render it sensitive to even slightly higher concentrations during FRDA disease progression (Koeppen et al., 2007). Tissue atrophy of the CDN and DRG likely occurs due to the terminally differentiated, non-mitotic nature of neurons, which therefore are unable to survive prolonged damage downstream from the loss of FXN. Clearly, proper mitochondrial physiology including fission/fusion, networking, oxphos, and DNA repair enzymes are essential (Golpich et al., 2017). Continual oxidative insult creates a decline in DNA repair machinery including base-excision repair (BER) and mismatch repair (MMR) and promotes mtDNA mutations (Leandro et al., 2015). mtDNA is susceptible to damage due to a lack of histone protection while it encodes 13 of the ETC complex proteins and some translational machinery; this genome loss compromises oxphos efficiency and mitochondrial homeostasis (Cooper, 2000). Loss of FXN is also associated with increases in mtDNA damage and lack of proper BER functionality (Bhalla et al., 2016). Thus, neuronal protection is essential in attenuating neurodegeneration in FRDA, achievable only *via* by a more detailed understanding of the cellular defects associated with this disease.

## Iron and FXN

Ferrous (soluble) and ferric (chaperone-protein bound) oxidation states of iron support its use in electron transfer as in the ISC in enzymes of the ETC, but this redox facility also supports the generation of reactive oxygen species (ROS) when unregulated ferrous iron undergoes the facile auto-oxidation that occurs at neutral pH. All four complexes of the ETC contain iron prosthetic groups: the first three contain various numbers of ISCs, and complex IV contains two heme groups. FXN deficiency leads to loss of function of all of these protein complexes, and endomyocardial biopsies of FRDA patients show decreases in complexes I–III (Rötig et al., 1997; Gonzalez-Cabo et al., 2005). This is particularly interesting as complex II is completely nuclearly encoded and thus, this effect cannot be due to increases in mtDNA damage. Increased mtDNA mutations have been identified in shRNA-mediated FXN-deficient fibroblasts, as well as mutations in the nuclearly encoded BER gene *NTHL1* (Bhalla et al., 2016). Importantly, some BER DNA-repair enzymes rely on ISC-catalytic centers, which becomes evident as FRDA fibroblasts stressed with hydrogen peroxide exhibit a knockdown of repair of damaged DNA bases in comparison with WT controls (Lukianova and David, 2005; Bhalla et al., 2016).

There are two forms of the aconitase enzyme: (1) mitochondrial, which is an essential enzyme of the Krebs cycle; and (2) cytosolic, which is an iron response protein (IRP) (Chappell, 1964). Thus, aconitase is an essential protein for both regulation of iron homeostasis and in energy metabolism (Kim et al., 1996). FRDA was described as a disease of metabolic dysfunction even from the early days of research when patient whole-blood analyses showed increased blood lactate, implicating a higher reliance on glycolysis due to deficiencies in oxphos (Finocchiaro et al., 1988). Endomyocardial biopsies from two FRDA patients were analyzed for total aconitase finding decreased expression in patients; similar findings have been corroborated in yeast models (Rötig et al., 1997; Foury, 1999). FRDA heart biopsies relative to the mean of controls show activity decreases in complexes I–III and aconitase. Also, FRDA patient skeletal muscle mitochondria have decreased activities of complexes II–IV with a marginal effect on aconitase and complex I (Rötig et al., 1997). Furthermore, free mitochondrial iron in FRDA fibroblasts damages mtDNA encoding ∼15% of the proteins in complexes I–IV (Bhalla et al., 2016). Mitochondrially encoded genes including NADH dehydrogenase MT-ND (subunit of complex I) and ATP synthase genes MT-ATP-6 and -8 are decreased in FRDA patient mitochondrial genomes (Singh et al., 2015). Impaired oxphos in FRDA patient platelets has also been demonstrated by lack of glucose incorporation into acyl-CoA; this decrease correlates to GAA expansion length (Worth et al., 2015). This metabolic defect may lead to upregulation of fatty acid β-oxidation, needed to maintain mitochondrial membrane potential and ATP production in the context of the FXN deficiency. The use of acetyl-CoA in fatty acid β-oxidation may be consistent with epigenetic findings in FRDA including lower histone acetylation and higher H3K9 methylation (Herman et al., 2006; Worth et al., 2015). Collectively, defective oxphos is pathophysiological in FRDA (Singh et al., 2015; Worth et al., 2015; Bhalla et al., 2016).

### Redox Chemistry in FXN Deficiency

The level of oxidative stress in FXN deficiency is fairly well documented although some *in vivo* reports are inconsistent with these *in vitro* biochemical findings, a difference that may reflect compensation by antioxidant activity in organ or whole-body systems (Martelli et al., 2012; Martelli and Puccio, 2014). However, FRDA patient–derived fibroblasts are, in fact, more susceptible to both iron- and hydrogen peroxide–mediated cell stress (Yang et al., 1999). A simple hypothesis for the onset of redox stress is the free iron accumulation due to loss of FXN’s iron chaperone function. Temporal manifestation of free cellular iron remains elusive, however (Lupoli et al., 2018). For instance, how long and to what extent does iron-mediated pathology challenge cells? Postmortem examination affords insight to the disease endpoints while progressive disease manifestation remains relatively undefined. Addressing this debate on the pathogenicity of ROS in FRDA can start with development of temporally focused research, likely best performed with inducible knockout animal models (Lupoli et al., 2018). The findings with respect to iron-mediated pathophysiology in FRDA are outlined below.

### Fenton Reaction Pathology in FRDA

**FIGURE 2:**
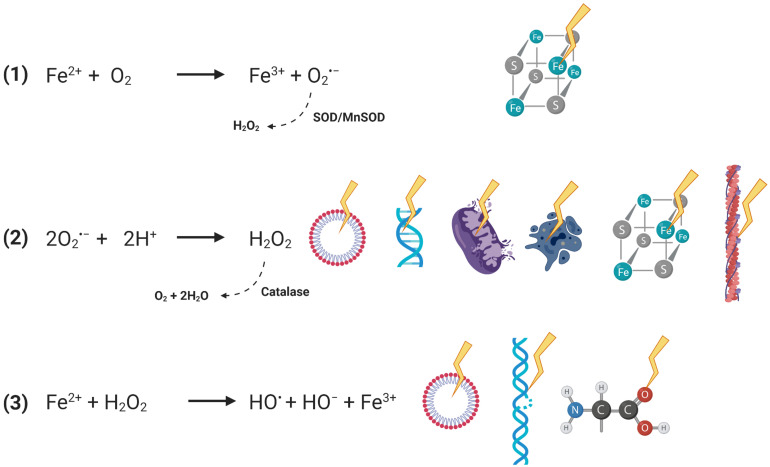
Stepwise demonstration of the Fenton reactions and subsequent pathological effects. Reactions depicted with dotted arrows represent reverse reactions mediated by cellular antioxidants. Production of superoxide radical anion (SORA) in the first reaction is exploited and secreted by immune cells for pathogenic control, indicated by the solid curved arrow. Pathological consequences include oxidation of susceptible iron atoms in [4Fe–4S]^2+^ clusters. Production of hydrogen peroxide in the second reaction is damaging to lipid bilayers, DNA, mitochondrial membrane potential, causes apoptotic cascades, damages ICS, and disrupts the actin cytoskeleton. Production of hydroxyl radical in the final reaction is similarly known to damage lipid bilayers, cause DNA strand breaks, and be reactive with all known biomolecules.

Mitochondria are the highest natural producers of ROS production in cells as an estimated 2% of electrons prematurely escape from the ETC complexes and react with molecular oxygen leading to the formation of hydrogen peroxide, a process termed “electron leak” (Brown et al., 2010). However, the production of ROS remains at tolerable physiological levels in cells capable of a commensurate antioxidant response. The Fenton reaction described in the late 1800s outlines the production of ROS due to single-electron reduction of molecular oxygen in the presence of free ferrous iron; this is illustrated in Figure 2, along with corresponding cellular targets and antioxidant mechanisms.

The first step describes the production of superoxide radical anion (SORA) which will readily oxidize iron atoms in [4Fe–4S]^2+^ clusters to the unstable [3Fe–4S]^3+^ form by extruding an iron atom; this renders the enzymatic center inactive while the released iron can support the Fenton chemistry redox cycle (Flint et al., 1993). Considering the multi-factorial reactivity of ROS, their presence in the cell has to be tightly managed. Superoxide species are readily converted to hydrogen peroxide by the superoxide dismutases (SOD); Sod1 is a cytoplasmic protein while Sod2 is found in the mitochondria. Importantly, Sod1 and 2 transcripts are found decreased in FRDA-model mice DRGs downstream from deficient antioxidant signaling, which is discussed further below (Shan et al., 2013).

Hydrogen peroxide is very membrane permeable and is taken up *via* aquaporins and thus has the potential to create global cell oxidative stress. In addition, H_2_O_2_ is also an apoptotic trigger with the activation of caspase-3 and cytochrome *c* release; peroxide induces loss of mitochondrial membrane potential, increases free intracellular calcium, and causes direct oxidation to lipids and DNA strand breaks (Hoyt et al., 1997; Bienert et al., 2006; Xiang et al., 2016). Similar to SORA, hydrogen peroxide inactivates ISCs by displacing an iron atom (Jang and Imlay, 2007). Peroxide is a diversely reactive species, for which catalase is the cell’s antioxidant defense, which induces its disproportionation into water and molecular oxygen (Chelikani et al., 2004). The pathological reactivity of the cytoskeleton with peroxide and its relation to FRDA cell pathophysiology is discussed in later sections.

The final step of Fenton chemistry leads to the hydroxyl radical, which is the most reactive and most potent oxidizing species. The hydroxyl radical damages all biomolecules, inducing lipid peroxidation and DNA strand breaks (Gutteridge, 1984; Halliwell, 1987; Fu and Dean, 1997; Balasubramanian et al., 1998; Zarkovic, 2020). The Fenton reactions highlight the intuitive centrality of iron’s potential pathophysiology to oxidative stress in FRDA. This still remains under discussion, although the available biochemical and cell biologic data render this molecular aspect of disease an important area of investigation.

#### Nrf2 Dysregulation in FRDA

Recently, defects in nuclear factor erythroid 2-related factor 2 (Nrf2) signaling have been identified in FRDA. Nrf2 under physiologic conditions exists as a cytoplasmic protein bound to Kelch-like ECH-associated protein 1 (Keap1), keeping Nrf2 ubiquitinated for lysosomal degradation (Itoh et al., 1999; Stewart et al., 2003). Importantly, a role of Keap1 is to tether Nrf2 to bundles of actin stress fibers which congregate in the cytoplasm at the periphery of the nucleus. Glutathionylation of Keap1 is responsible for the release of Nrf2 during times of oxidative stress, allowing for translocation of Nrf2 to the nucleus where it positively regulates expression of genes containing antioxidant-responsive elements (AREs) (Kensler et al., 2007; Paupe et al., 2009; Carvalho et al., 2016).

shFXN neuron-like neuroblastoma SKAN cells are deficient in Nrf2 signaling in oligomycin or t-butyl hydroquinone (*t*BHQ) oxidative stress models (Paupe et al., 2009). Oligomycin inhibits ATP synthase and promotes mitochondrial superoxide leakage while *t*BHQ promotes hydrogen peroxide production. Treatment dysregulated Nrf2 cytoplasmic and nuclear localization and its downstream signaling in the FRDA cells, as discussed later. The F-actin cytoskeleton was highly disorganized, preventing Nrf2 binding. Furthermore, Nrf2 could not properly localize to the nucleus of FXN-deficient cells, thus preventing its activity as a transcription factor (Paupe et al., 2009).

This is puzzling considering that Nrf2 is active when unbound to Keap1 and actin. However, this may be due to grossly disorganized actin fibers in the FRDA model. Unlike WT control cells that dock Nrf2 in close proximity to the nucleus, FXN-deficient cells had stress fibers organized toward the cell periphery. Thus, a total re-organization of the cytoskeleton may be compounding impaired Nrf2 activity by challenging its trafficking to the nucleus. Clearly, a deficiency in Nrf2 signaling in FRDA exhibits the well-documented hypersensitivity to oxidative stress, a phenotype with co-incident cytoskeletal dys-organization. Actin dynamics are strongly hindered by glutathionylation as discussed later which could be either a cause and/or effect of altered Nrf2 signaling. This may also be considered a feedback mechanism—does altered Nrf2 signaling prevent proper actin organization or does this improper actin organization result in deficient Nrf2 signaling? Such questions remain.

Examination of the antioxidant capacity downstream of Nrf2 signaling in FXN-deficient cells in basal conditions revealed a marginally high and consistently elevated total SOD levels, but a decrease in the ratio of reduced glutathione to oxidized glutathione (GSH/GSSG-P) (Paupe et al., 2009). Hand in hand with elevated SOD levels was an observed small but consistent rise in intracellular hydrogen peroxide levels. This increase in SOD and decrease in GSH indicate that FXN-deficient cells need to consistently deal with a heightened state of oxidative stress. FRDA fibroblasts treated with exogenous stress to induce the Nrf2 response indeed could not induce proper antioxidant compensation including upregulation of catalase, NQO1, and GST-P1 after *t*BHQ treatment or catalase, GST-P1, and SOD2 after oligomycin treatment. The same experiment in the neuron-like FXN-deficient SKAN cells demonstrated a lack of NQO1 or Grx response after *t*BHQ treatment or NQO1, Grx, and GST-P1 increase after oligomycin treatment. Significantly, the same phenotype of altered localization and signaling of Nrf2 was seen in the FXN-deficient SKAN cells. Euk134, a catalase mimetic, restored Nrf2 signaling in FRDA cells, consistent with the model that an antioxidant deficit is comorbid in FRDA (Paupe et al., 2009).

Thus, these several data highlight the knockdown of the normal antioxidant response in FRDA particularly with respect prolonged redox stress, and coincide with the earlier reports of cytoskeletal abnormalities reported in FXN-deficient cells (reviewed below). Nuclear translocation and induction of antioxidant enzyme expression was restored in the presence of Euk134, but not in the presence of iron-chelating drugs deferiprone or desferrioxamine, indicating that the molecular defects in FRDA extend beyond the Fenton reactions *per se* and likely are due partially to impaired antioxidant signaling (Paupe et al., 2009). These findings add to the model that FRDA is a disease of production of oxidative stress, suggesting that FRDA is a disease also of deficient response to oxidative stress. This is particularly important considering that deferiprone and desferrioxamine iron chelation drugs were among the first therapeutics used in FRDA patients, albeit with minimal benefit.

## FXN and Ferroptosis

Ferroptosis has long been regarded as an apoptotic response to cell iron overload, a cell pathology linked to the Fenton reaction-dependent oxidative stress noted earlier. Ferroptotic cell death could explain the “dying back” phenotype of the large neurons of the CDN and DRG observed in postmortem analyses (Said et al., 1986; Koeppen and Mazurkiewicz, 2013). Furthermore, ferroptosis in FRDA patient neurons may explain the brain iron deposition hypothesis, where ferroptotic neuron death has the potential to release iron in the interstitium for local absorption by microglia and astrocytes (Koeppen et al., 2007). This may indicate iron deposition as a secondary pathological manifestation though largely confined to advanced stages of disease considering its progressive accumulation (Ward et al., 2019). This controversy regarding temporal pathological consequences in FRDA would clearly benefit from a more detailed understanding of the molecular basis and progression of CNS cellular pathology.

A model of the ferroptotic phenotype is provided by FXN-suppressed fibrosarcoma cell lines that exhibit ferroptotic death (Du et al., 2020). In this model, apoptosis and necrosis were ruled out as ferroptosis inhibitors Ferrostatin-1 and GSH could rescue cell death but caspase inhibitors for apoptosis (Z-VAD-FMK) and necrosis inhibitor (IM-54) could not. Both treatment with ferritin heavy-chain and ectopic FXN rescued iron overload and inhibited ferroptotic death; the latter treatment also increased mtDNA copy number (Du et al., 2020). This demonstrates that the presence of FXN prevents iron overload–mediated ferroptotic cell death. Demonstration of ferroptosis in FRDA provides the framework of a mechanism of cell death in the fibrosarcoma cell line, but the question remains as to the impact of the chronic oxidative stress resulting from loss of FXN and what pathological implications this may have *in vivo*. Thus, ferroptosis may be the end result of cellular defects in FRDA after cumulative oxidative insult.

A recent paper also implicates Nrf2 signaling in ferroptotic resistance in hepatoma cells (Sun et al., 2016). Ferroptosis was induced by the addition of erastin, sorafenib, or buthionine sulfoxamine (BSO), either directly inducing a ferroptotic cascade (erastin) or inducing oxidative stress and lipid peroxidation for upstream activation (sorafenib, BSO). Interestingly, Nrf2 activity was modulated not at the mRNA level but at the protein level during ferroptotic induction, and the half-life of the protein was increased with erastin treatment. In the absence of Nrf2, cells were pushed more to ferroptosis with the additional phenotypes of lipid ROS peroxidation, GSH depletion, and increased cell iron levels. This finding was corroborated in an *in vivo* Nrf2-KD mouse model treated with the same ferroptotic inducers. Cell death increased as indicated by the fact that tumor size of Nrf2-deficient mice was significantly smaller compared with WT (Sun et al., 2016). This implicates Nrf2 signaling and its downstream proteins as negative regulators of ferroptosis by circumventing cell growth inhibition (Paupe et al., 2009; Sun et al., 2016). With loss of Nrf2 signaling in FRDA, FXN-deficient cells have a heightened sensitivity to ferroptotic cell death. Ferroptosis is a widely acknowledged and seemingly intuitive method of cell death in FXN deficiency, and could be responsible for neuronal death. This model supports cell death resulting from cellular iron accumulation, but the extent of previous cellular homeostatic deterioration, e.g., from oxidative stress, remains elusive.

## Redox Stress, Glutathione, and Cytoskeletal Breakdown in FRDA

With the known oxidative stress profile in FRDA, many reports have investigated cellular compensation by antioxidant responses. Early understanding of glutathione metabolism indicated abnormalities in neurodegenerative diseases and preliminary work on the redox environment of FRDA patients indicated heightened oxidative stress biomarkers including blood malondialdehyde and urine 8-hydroxy-2-deoxy-guanosine (8-OHdG) (Emond et al., 2000; Schulz et al., 2000). This prompted further investigation of glutathione metabolism in the context of redox stress in FRDA patients (Piemonte et al., 2001). In contrast, other markers of oxidative stress such as urinary F_2_-isoprostanes have failed to show differences among FRDA patients and healthy controls, or present a correlation with GAA expansion, patient age, disease stage, or use of antioxidants including vitamin E, coenzyme Q10, or idebenone (Myers et al., 2008). Isoprostanes are a specific marker of lipid peroxidation *in vivo* and thus should reflect iron-mediated ROS and depletion of GSH levels. As previously mentioned, the relevance of ROS in FRDA is strongly supported in *in vitro* models but poorly replicated in some physiologic contexts, dichotomies yet to be resolved.

Glutathione exists in either the free, reduced state (GSH), free oxidized state (GSSG), or its “active” oxidized, protein-bound counterpart (GSSP) by forming a disulfide bond to a cysteine residue. GSH is the form which is redox active in combating oxidative stress. The enzymatic cycling of glutathione depends on the activities of glutathione peroxidase (Gpx), glutaredoxin (Grx), and the cellular redox potential set by NADP/NADPH. During redox stress when NADPH is lacking, the oxidized forms of glutathione—GSSG or GSSP—are unable to be cycled back to GSH through the activity of Grx and thioredoxin (Trx) which reduce GSSG to 2GSH or disulfide bonds, respectively. In resting cells, the GSH/GSSG ratio is ∼100:1 whereas in oxidative stress it can fall as low as 1:1 (Chai et al., 1994). Whereas the reduction potential of the resting cell is ∼−250 mV, in severe oxidative stress it will rise by as much as 100 mV depending on the absolute concentration of GSH (Schafer and Buettner, 2001).

Hydrogen peroxide treatment of isolated mitochondria increases the presence of oxidized glutathione at the expense of its reduced form and a loss of presence of reduced pyridine nucleotides could not rescue those levels (Garcia et al., 2010). However, NADH and NADPH can be recovered by supplementing the isolated redox stressed mitochondria with ETC complexes I and II. Prevention of oxidative stress buildup upon ETC shutdown is due to the increased prevalence of NADH, which in the presence of NADP+ and nicotinamide nucleotide transhydrogenase can form NADPH for reducing equivalents in oxidative stress repair reactions (Garcia et al., 2010; Adler et al., 2014). Production of NADH by the Krebs cycle is essential to supply of ETC complex II, and thus, the mitochondrial energy production pathways are important in providing substrates for the buffering of cell redox potential. Thus, Garcia et al. (2010) demonstrated that proper mitochondrial function helps to indirectly maintain glutathione metabolism through production of reduced respiratory substrates.

Glutathionyl-hemoglobin is recognized as a marker of oxidative stress and thus was examined in the blood of FRDA patients in an early report on disease pathology (Piemonte et al., 2001). Although the total amount of glutathione was not different among the FRDA and control samples, there was a reduction in free glutathione in disease, indicating a more oxidative environment and heightened GSSP species. The reliance on NADPH in normal GSH/GSSG-P cycling makes this ratio a marker of mitochondrial stress and suggests one aspect of FRDA cellular pathology. Analysis of glutathione in patient blood samples stimulated a plethora of subsequent research on *in vitro* glutathione homeostasis.

Following the Piemonte et al. (2001) findings of abnormal glutathione metabolism in FRDA was the Pastore et al. (2003) identification of glutathionylated actin in patient isolated fibroblasts. FRDA cells again exhibited increased GSSP species compared with control fibroblasts whereas the addition of exogenous GSH rescued the ratio of GSH/total cell glutathione and improved cell viability. By both HPLC and co-immunoprecipitation, oxidized glutathione was found to be linked to actin in the diseased cells. Examination at the whole-cell level revealed higher side scatter in cytometric analyses, indicating differential internal organization and structural complexity. Phalloidin actin staining was attenuated in the FRDA cells relative to controls, revealed disorganization of filamentous structures, and demonstrated changes in cell size and shape (Pastore et al., 2003). Again, this was a phenotype that could be rescued with the addition of reduced glutathione. Glutathione of course has a broad target range; however, actin glutathionylation is the most common transient modification with respect to the cytoskeleton upon oxidative stress (Dalle-Donne et al., 2003; Townsend, 2007).

To link iron-mediated oxidative stress to cytoskeletal alterations, Pastore et al. (2003) treated control fibroblasts with iron (II) sulfate to induce oxidative stress. The treated cells indeed reflected changes in glutathione of FRDA cells; a decrease in the GSH/GSSG-P ratio and increased glutathionylated actin. Unfortunately, changes to cell morphology that reflect the phenotype of FRDA could not be replicated with iron stress alone, indicating a multi-faceted onset of pathophysiology in FRDA. Previously mentioned in the context of the Fenton reaction was the disruption of the actin cytoskeleton by hydrogen peroxide. Hydrogen peroxide has been found to alter actin organization and filament polarization in both bovine aortic endothelial cells and yeast. In the former cells, peroxide in a time- and concentration-dependent manner disrupted actin filament organization that correlated with increased permeability, endocytosis, and transcytosis by promotion of cytoskeletal reorganization (Liu and Sundqvist, 1995). Peroxide treatment in yeast changes actin staining from its normal branched organization into puncta formation, creating what Farah et al. (2011) call “oxidation-induced actin bodies”. These studies suggest changes to normal actin physiology downstream from iron-mediated redox stress and altered antioxidant response in FRDA.

Subsequent investigation has identified that prolonged redox-mediated glutathionylation of actin monomers can be pathological (Chen and Ogut, 2006). This prevents both proper F-actin elongation, polymerization, and binding affinity to tropomyosin, a stabilization factor of the cytoskeleton (Dalle-Donne et al., 2003; Gagat et al., 2014). This is unfortunately the extent of knowledge for glutathionylated proteins in FRDA or FXN deficiency and clearly represents an essential focus of future research into the molecular basis of FRDA disease comorbidities. One clear example of a research focus is Nrf2: Nrf2 is activated by glutathione, but is inactive in FRDA. Is this latter phenotype a key element in FRDA cell sensitivity to oxidative stress?

Cysteine residues of actin are sensitive to oxidative stress, and Cys374 is the most often glutathionylated (Tsapara et al., 1999). Cys374 is the penultimate cysteine residue at the C-terminal end of actin monomers and is essential in the promotion of inter-monomer communication in filament formation. A deficiency in polymerization with GSS-actin was identified specifically during elongation, the determinant of filament polymerization and branching capacity (Dalle-Donne et al., 2003). Dalle-Donne et al. (2003) described three actin dynamics affected by glutathionylation: (1) slower rate of polymerization, (2) less efficient polymerization, and (3) a lesser extent of polymerization. Thus, F-actin deficiency results from glutathionylation not as an inhibition and complete loss but instead by slower rate of formation. Treatment with the reducing agent DTT rescues actin polymerization capacity, indicating actin glutathionylation is reversible and does not induce protein denaturation. Glutathionylated actin has also been found to exhibit decreased mechanical stability and disrupt more easily than its un-modified form in the presence of mechanical shear stress (Stournaras et al., 1990). Thus, glutathionylation of actin is a pathological consequence of increased oxidative stress. This aspect of FRDA cell physiology is unexplored as a potential therapeutic target.

This gap in knowledge raises the question of the pathological implications of actin dysregulation in different cell models. Actin is the basis for cell junctional anchorage and is the driving force for motility and contractility. In addition, actin dynamics are driven by ATP whose synthesis is compromised by run-down of oxphos in FRDA. Indeed, the literature focusing on actin dynamics in FRDA patient fibroblasts includes no data on cellular contractility or motility. Clearly, this degradation of cytoskeletal function is ubiquitous in FRDA given that both FXN and actin are ubiquitously expressed, essential, and that the repeat expansion in FXN accrues throughout successive rounds of cellular replication. Nonetheless, these reports of two decades ago and the description of PIP5K1β in the following section reflects the extent to which cytoskeletal alterations have been noted as hallmarks of this progressive, fatal disease.

### Other Cytoskeletal Abnormalities in FRDA

Actin’s dynamic nature in cells is responsible for its homeostatic role in signal cascades, organelle distribution, junctional integrity, contractility of motile cells, and cell shape. Thus, actin dynamics are essential to proper cell homeostasis and should be thoroughly investigated in the context of FRDA pathophysiology. After a decade-long gap in literature of the cytoskeleton in FRDA, Bayot et al. (2013), who reported on physical alterations to DNA sequences resulting from FXN GAA expansions, noted transcriptional silencing and decreased protein production of PIP5K1β, an isoform of PIP5K. PIP5K1β is encoded just upstream of FXN on human chromosome 9. PIP5K is activated by signaling from RhoA/ROCK for modulation of actin dynamics through phosphorylation of PI(4,5)P_2_ (reviewed extensively by Van den Bout and Divecha, 2009). In FRDA patient–derived fibroblasts, a lack of PIP5K1β prevented efficient phosphorylation of PI(4,5)P_2_, leading to destabilization of F-actin. Consequently, when the FRDA fibroblasts were lifted and allowed to settle, there was decreased spreading, indicating deficient contractility and motility due to this cytoskeletal disarrangement (Bayot et al., 2013). Another gene flanking FXN in both humans and mice encodes tight junction protein 2 (TJP2/ZO-2). Bayot et al. (2013) find no alterations to the expression of TJP2 resulting from FXN mutation, but otherwise, there has been little experimental consideration of this possible co-morbid genetic insufficiency.

In relation to the previously described alterations to mitochondrial function in FRDA, mitochondrial transport along neuronal axons and dendrites is mediated by interactions with cytoskeletal microtubules and neurofilament illustrating a key link between actin, mitochondrial function, and neuronal homeostasis (Sparaco et al., 2009; Bürk, 2017). Alterations in axonal transport are implicated in drosophila and mouse models of FRDA which may provide insight on neurodegeneration (Shidara and Hollenbeck, 2010; Mollá et al., 2016). Clearly, the ubiquitous and essential nature of actin renders glutathione modification an important area for further examination. Aside from the work on the glutathione-mediated actin dysregulation in FRDA and the recently published paper on PIP5K, little has been reported on the cytoskeletal dynamics in FRDA. Also, there has been little attempt to link actin modifications in FRDA to a deficiency of PIP5K1β or the onset of oxidative stress and the resulting protein glutathionylation, what would constitute a mechanism that would amplify the downstream effects of a FRDA-related oxidative stress. These are likely to be significant deficiencies in cell physiology that are linked to this genetic disorder.

## Mitochondrial and Cytoskeletal Stress, and Barrier Breakdown

As mitochondria are central to efficient energy production in all cell types, optimal function is necessary for normal cell homeostasis. With the preceding sections focusing on cytoskeletal network disassembly downstream of FXN deficiency, it is important to note that mitochondrial stress in itself is pathological in cytoskeletal dynamics. Barrier-forming cells are highly reliant on the strength of their cytoskeleton, largely on actin, as it anchors the cytoplasmic scaffolding tight junction (TJ) proteins that maintain paracellular impermeability. Here, the importance of mitochondrial maintenance in cytoskeletal and junctional barrier integrity is highlighted.

The first report to examine the importance of mitochondrial maintenance in barrier function quantified blood–brain barrier integrity when treated with mitochondrial inhibitors (Doll et al., 2015). With the mitochondria being the most efficient method of energy production, it seems intuitive that proper functional is essential to cell and tissue maintenance. In an *in vitro* model system, LPS treatment of the bEnd.3 murine brain capillary endothelial blood–brain barrier (BBB) model decreased maximal respiration and spare respiratory capacity, indicating that inflammatory stress suppresses oxphos capacity. This treatment also decreased expression of multiple protein subunits of the ETC. Furthermore, LPS treatment *in vivo* in mice increased brain leukocyte extravasation consequent of a leaky BBB (Doll et al., 2015). These studies demonstrate increased barrier permeability resulting from decreased mitochondrial function and oxidative stress. Clearly, these studies relate to FRDA with respect to the profile of oxidative and mitochondrial stress but do not demonstrate the BBB permeability is co-morbid with FRDA, also this analogy does indicate that further research is required to see if similar mechanisms of barrier dysregulation could occur in FRDA models.

To demonstrate mitochondrial essentiality in barrier formation, bEnd.3 cells were cultured with drugs that selectively inhibit different respiratory complexes. Increased barrier permeability was demonstrated with heightened paracellular flux of FITC-dextran and examination of junctional proteins by immunohistochemistry (Doll et al., 2015). Clear disturbances in TJ protein ZO-1 continuity were visualized in the mitochondrial stress model; ZO-1 is the cytoskeletal scaffold required for junctional actin anchorage. Doll et al. (2015) strongly argued that the mitochondrial maintenance for barrier integrity is poorly appreciated in the literature and has broad implications in the therapy of several neurodegenerative disorders. Importantly, none of their experimental stress models showed increased cell death which indicates that the observed junctional dysfunction results from increased paracellular permeability rather than cell death (Doll et al., 2015).

A linkage between the cytoskeleton and bioenergetics of the cell has come to light also in reports of bovine capillary cells with dysregulated F-actin resulting from stress of the mitochondria and energy production crisis (Tornabene et al., 2019). These barrier cells were nutritionally starved with simultaneous oxygen and glucose deprivation and exhibited increased permeability after actin dysregulation. Considering the role of FXN in energy metabolism, respiratory starvation could also add to the cytoskeletal pathology in FRDA. Thus, the link between metabolic maintenance and cytoskeletal function while not currently in the FRDA spotlight has been suggested in research unrelated to the pathophysiology in FRDA. The aforementioned work by Doll et al. (2015) and Tornabene et al. (2019) underscore the interplay of oxidative stress, mitochondrial physiology, and cytoskeletal function, while focusing on barrier cell types which are heavily reliant on the cytoskeleton. The current review serves to emphasize this point in the context of FRDA and other NBIA diseases.

These studies on mitochondrially stressed endothelial cells pose the question: do barrier cells deficient in FXN similarly lose their impermeability upon mitochondrial stress (Doll et al., 2015; Tornabene et al., 2019)? Among important barrier cells are brain microvascular endothelial cells (BMVECs), the primary cell type forming the BBB. Underlying support is maintained by astrocytes and glia, but the tight junctions (TJs) between adjacent BMVECs maintain the selective solute impermeability necessary for separation of blood and the brain interstitium. A loss in cytoskeletal integrity arising from dysfunctional metabolic pathways in FRDA could be contributing to alterations to other types of barrier-forming cells such as neuronal types [blood–retinal barrier, blood–dorsal root ganglion barrier (B-DRG)] and non-neuronal types (intestinal epithelial cells, cardiac vascular cells). Interestingly, the B-DRG barrier is outside of and leakier than the BBB, so its upkeep must be essential to protect this structure which is already susceptible in FRDA. Investigation of general vascular barrier systems clearly are of interest in FRDA as it relates to CNS pathology relative to the cytoskeletal abnormalities first identified in patient fibroblasts. Concerning the defects in contractility and motility in PIP5K1β/FXN-deficient cells, special consideration may also be placed on circulatory lymphocytes which rely on actin stress fiber contractility for proper motile forces. The focus of this review has been to interrogate previous findings of oxidative stress–mediated cytoskeletal alterations relative to other cell types. Further investigation may prove or disprove a connection between the cytoskeleton as related to the NBIA co-morbid in FRDA.

A more thorough insight on the cytoskeletal maintenance of barrier-forming cells in FRDA may provide key insight into neurodegeneration specifically if the CDN iron deposition results from BBB leakiness, and if it contributes to neurodegeneration. Examination of cytoskeletal homeostasis in other tissue and barrier systems may answer the question of tissue specificity in disease—i.e., if disease severity is also correlated to tissues highly reliant on cytoskeletal integrity, stiffness, shape, structure, elasticity, etc. Thus, this review is aimed at re-highlighting the importance of the cytoskeleton in FRDA which has been largely underappreciated over the past decade.

## Therapeutic Strategies in FRDA

### Current Therapeutic Strategies: Managing Iron and Oxidative Stress

Although current therapeutic options strategically include iron chelators and antioxidant drugs, there is no optimal treatment for FRDA at this time. The classically prescribed iron chelators are desferrioxamine (DFO) and deferiprone (DFP). DFO is impermeable to the mitochondrial membrane and thus fails to redistribute the accumulated iron between sub-cellular compartments (Ponˇka et al., 1979). In addition, through its effect on the RNA-binding capacity of the iron response element binding protein (IRP), DFO decreased FXN transcription and protein production in FRDA cells (Li et al., 2008). On the other hand, DFP is permeable to the mitochondrial membrane and is superior in mobilizing iron between sub-cellular compartments and for donation to pre-erythroid cells for hemoglobin synthesis (Sohn et al., 2008). DFP is effective in decreasing the CDN-specific iron concentrations after 6 months of treatment in FRDA patients in its initial clinical trials, indicating that cerebellar iron is redox active and chelatable (Boddaert et al., 2007). Initial reports seemed promising based on the drug’s mechanism of action but at its effective concentration, DFP had adverse effects in a FXN-deficient neuroblastoma cell line on cell proliferation and decreased aconitase activity, and worsened a subset of patient ataxic gaits in clinical trials (Boddaert et al., 2007; Goncalves et al., 2008; Pandolfo and Hausmann, 2013). Iron chelation is a tricky balancing act to navigate as it may adversely affect Fe–S cluster formation or even further deplete normal iron concentration in the brain. Iron plays an essential role in all tissue types and must remain at tolerable, although bioavailable, concentrations.

Idebenone was another obvious choice for therapeutic benefit as its mechanism of action is as an antioxidant by scavenging free radicals. Idebenone proved to be cardioprotective and mildly delayed mortality in a cardiac-specific FXN Cre-lox mouse model, but it failed to rescue ISC functionality (Seznec et al., 2004). Combination of idebenone with DFP in FRDA patients reduced iron loading in the CDN and improved cardiac defects, but the combination worsened patient ataxic gait, increased the risk of neutropenia, and reduced serum iron (Velasco-Sánchez et al., 2011). Failure of these intuitive drug therapies underlie the complicated nature of FRDA etiology.

#### Experimental Techniques: Antioxidants, Nrf2 Stimulation, and Epigenetics

Experimental techniques which combat oxidative stress include the following: peroxisome proliferator activated receptor gamma (PPARγ) coactivator 1-alpha (PCG1-α), erythropoietin, RTA-408-Omaveloxolone, dimethyl fumarate (DMF), sulforaphane, *N*-acetyl cysteine (NAC), and EPI-743. The discussion of antioxidant drugs is followed by a discussion of potential epigenetic targets in FRDA using the histone deacetylase inhibitor 109/RG2833. Information on emerging drugs and clinical trial information can be found on the Friedreich’s Ataxia Research Alliance website (curefa.org).

PCG1-α is a regulator of mitochondrial biogenesis and antioxidant-targeted transcription factor whose expression is positively correlated to levels of tissue energy metabolism (Gureev et al., 2019). Knockdown in murine mesenchymal stem cells and PCG1-α null-mouse neurons blunts proper SOD enzyme expression (already reduced in FRDA), consistent with findings of PCG1-α downregulation in FRDA fibroblasts (Jiralerspong et al., 2001; Marmolino et al., 2010; Shan et al., 2013). Consequently, PCG1-α null mice succumb more readily to 1-methyl-4-phenyl-1,2,3,6-tetrahydropyridine (MPTP) and kainic acid neurodegenerative inducers, suggesting PCG1-α stimulators as FRDA therapeutics (St-Pierre et al., 2006).

Erythropoietin (EPO) is a hematopoietic signaling factor for survival and differentiation which increased FXN levels in FRDA patient lymphocytes, mobilizes iron to heme, and exhibits some neuro- and cardioprotective capacity, suggesting its use in clinical trials (Boesch et al., 2007). EPO treatment in FRDA patients increased lymphocyte FXN levels, reduced levels of 8-OHdG back to baseline, and promoted neurological score improvement over 8 weeks (Boesch et al., 2007). However, challenges in follow-up studies include reaching statistically significant increases in FXN expression, changes in blood-iron levels, defining proper dosage amounts, or proving any clinical benefit, thus complicating its otherwise rational application in FRDA (Boesch and Indelicato, 2019).

RTA-408/Omaveloxolone slows Nrf2 proteolysis, proving to restore otherwise blunted complex I activity in FRDA mouse model cerebellar granule neurons and FRDA patient fibroblasts (Strawser et al., 2017; Abeti et al., 2018). It was further cryoprotective in fibroblasts by preventing mitochondrial membrane depolarization. This effect was not reported for the mouse model, which may reflect discrepancies in compensatory homeostatic mechanisms present in whole-body systems, and highlights the complex, multi-factorial pathology of FRDA, leading to disparate findings in *in vitro* biochemical and *in vivo* experimental paradigms. In total, current drug therapeutics have strayed from the traditional iron chelators to more broad antioxidant agents or for supplementation of FXN levels.

DMF is an FDA-approved drug which stimulates Nrf2 activity by alkylating cysteine residues of Keap1, effectively inhibiting Nrf2 degradation and allowing for nuclear translocation. DMF is a widely reactive electrophile and thus modulates many protein thiols, which may have adverse effects on patient immunity and GSH presence (reviewed in Satoh and Lipton, 2017). Its metabolite, monoethyl fumarate (MEF) is less reactive and shows specificity to Cys151 of Keap1, thus preventing off-target effects on glutathione, but providing a less robust promotion of Nrf2 levels. Such tradeoffs of this drug classification render it a balancing act for use in patients. However, Jasoliya et al. (2019) have shown that FRDA-derived patient lymphoblasts and FRDA mouse models exhibit a significant increase in FXN transcription in a dose-dependent fashion after treatment with DMF. Furthermore, blood samples collected from multiple sclerosis (MS) patients treated with DMF showed significantly increased FXN levels after 3 months of treatment from their baseline levels. In the FRDA-lymphoblast cell line, treatment with DMF shows increased transcription initiation and procession following GAA expansion tracts, as well as increased mtDNA copy number (Jasoliya et al., 2019). This drug is already on the market and proves beneficial in other neurodegenerative such as relapsing MS, and may prove beneficial in the treatment of FRDA patients considering both its upregulation of FXN and of Nrf2 by Keap1 modulation.

In FRDA patient fibroblasts, Nrf2 is significantly upregulated at both the mRNA and protein levels by treatment with sulforaphane (Keap1 oxidant), DMF, NAC (L-cysteine pro-drug), EPI-743 (mitochondrial metabolism promotion and glutathione synthesis), idebenone, and omaveloxolone (Petrillo et al., 2019). Idebenone and omaveloxolone were the only two drugs tested that could not stimulate FXN levels, whereas NAC, EPI-743, and idebenone could not rescue GSH levels. This leaves sulforaphane, DMF, and NAC as promising drugs targeting the Keap1/Nrf2 axis. DJ-1 is a protein which is inactivated upon glutathionylation, which only NAC rescued in FRDA models, presenting this as a desirable treatment method. However, treatment of cardiac/skeletal muscle FXN-knockout mice with NAC actually decreases Nrf2 levels and proves no benefit in development of cardiomyopathy (Chiang et al., 2020). FRDA *Drosophila* models show that NAC benefits locomotor capacity, resistance to oxidative stress, survival capacity, and levels of aconitase (Russi et al., 2020). In line with discrepancies between models in FRDA research, reports of NAC in patient use do not provide strong evidence of benefit, nor the use of consistent dosages, and peer review (described in Pandolfo, 2008). On the other hand, NAC in experimental models of T-cell HIV infection restores GSH and increases the rate of GSSP species recycling (Ghezzi et al., 2002). GAPDH was further glutathionylated in this model upon cell treatment with diamide, reducing its activity to 25% of its baseline. Deglutathionylation by NAC partially restored its activity. This indicates that NAC is a drug with a wide spectrum of activity and may be beneficial in general contexts of redox restoration.

Another logical drug modality is the inhibition of protein–protein interactions of Nrf2 and Keap1 to prevent degradation. This method reduces the risk of off-target effects, although they are less common than drugs like DMF and MEF which act specifically on Keap1. Screening and development of small molecules to inhibit the binding sites of the two proteins are topical not only in the context of FRDA but also in other neurodegenerative and inflammatory diseases (reviewed in Abed et al., 2015).

Combating repressive chromatin marks and epigenetic FXN silencing, histone deacetylase inhibitor 109/RG2833 treatment in a neuronal cell model increases both FXN mRNA and protein levels concomitant with increased H3K9 acetylation in patient clinical trials (Soragni et al., 2014). Other epigenetic modifying techniques target the formation of complex DNA–RNA structures formed consequent of large expansion tracts. Synthetic nucleic acid probes complementary to expanded tracts recruit endogenous silencers and thus circumvent the transcriptional inhibition associated with GAA expansion, stimulating both transcript and protein abundance (Li et al., 2016). Recent advances in gene therapy have prompted investigation for FRDA patients by FXN supplementation. An *in vivo* mouse model of skeletal/cardiomyocyte FXN deletion rescued with gene therapy partially prevented and reduced the severity of pre-existing cardiomyopathy (Perdomini et al., 2014). However, this method of FXN rescue would not be beneficial for non-mitotic cells such as CNS neurons. This highlights the diverse nature of tissues affected in FRDA and the complex requirements for proper therapeutic drugs.

### Proposed Therapeutic Strategies: Targeting Oxidative-Stress Alterations in Cell Thiol Metabolism

With the focus of this review revolving around improper glutathione metabolism in FRDA, this section outlines potential therapeutic avenues for glutathione homeostasis, which remains to be thoroughly mined as a therapeutic avenue. As the Piemonte et al. (2001) and Pastore et al. (2003) papers demonstrate, exogenous GSH addition rescues the cytoskeletal abnormalities. This may have implications with respect to the antioxidant response because actin dysregulation was found to be inhibitory to Nrf2 signaling (Paupe et al., 2009).

Potential therapeutic targets of the glutathione pathway are Grx or sulfiredoxin (Sfx) that support reduction of GSSG/P into 2GSH. Sfx uses its antioxidant capacity to re-activate peroxiredoxin (Prx), which becomes inactive upon over-oxidation during redox stress. Prx is a thiol-containing antioxidant that can reduce hydrogen peroxide into two molecular equivalents of water. Supplementation of Sfx modulates antioxidant capacity of Prx. Furthermore, Sfx over-expression in HEK293T cells inhibits glutathionylation of actin even when treated with the pro-oxidant compound PABA/NO, a protein-glutathione initiator (Findlay et al., 2006; Hutchens et al., 2011). Consistent with the activity of Sfx in Prx signaling, HEK293T-Srx overexpression cells were more resistant to hydrogen peroxide–induced stress. This may be of interest in FRDA therapy because both peroxide and glutathione are important in the molecular pathophysiology of disease.

Supplementation of Grx may also be of interest for glutathionylated actin as seen in ROS-stressed neutrophils (Sakai et al., 2012). Actin dynamics are essential to neutrophil physiology as actin polymerization at the “leading edge” of these migratory phagocytes is responsible for motility following immune activation. NADPH oxidase (NOX) is an enzyme functional in many types of leukocytes catalyzing the production of SORA, which are sequestered in leukocytes to damage the membranes of invading pathogens. NOX activity was found to increase actin glutathionylation in neutrophils which was reversed with both DPI, an inhibitor of its activity, and DTT for reduction of the GSSP species (Sakai et al., 2012; Salamah et al., 2019). This indicates that modulation of oxidants proves beneficial in prevention of actin glutathionylation. Consistent with this pattern, Sakai et al. (2012) found that siRNA-mediated disruption of Grx increased actin glutathionylation and decreased polymerization in neutrophils, further reducing their chemotactic capacity, polarization, adhesion, and phagocytosis. This was true also in Grx-deficient murine neutrophils which had less chemotactic capacity for migration to the site of infection and were less bactericidal. Thus, extensive actin glutathionylation can be pathological in many different cell types, but stimulation of the glutathione enzymatic pathway such as supplementation of Grx may induce physiologic actin dynamics after their dysregulation as in FXN deficiency.

FRDA is not the only neurodegenerative disease which produces ROS stress in patients. This also has been identified in Alzheimer’s dementia (AD), HD, Parkinson’s disease (PD), and schizophrenia patients (Paul and Snyder, 2019). Accordingly, the search for BBB-permeant therapeutic drugs is ongoing. Cysteamine (CSEA) and its oxidized form cystamine (CSA) counteract oxidative stress and inflammation and also upregulate protective signaling through brain-derived neurotrophic factor (BDNF) in a mouse Parkinson’s disease (PD) model and Nrf2 transcription in the CNS (Borrell-Pagès et al., 2006; Calkins et al., 2010; Paul and Snyder, 2019). Treatment with CSEA/CSA increased signaling of both pathways and offset neurodegeneration in models of 3-NP lesion (preventing complete ETC complex II activity, giving striatal neurodegeneration), HD, PD, stroke, and schizophrenia (Paul and Snyder, 2019). CSEA/CSA works through the same mechanism of action as NAC (as a prodrug to cysteine) and therefore provides a component required for glutathione synthesis.

CSA treatment of a PD mouse model showed multiple molecular improvements associated with PD pathology including oxidative stress and mitochondrial physiology (Stack et al., 2008). PD shares several pathologic aspects with FRDA including neurodegeneration, mitochondrial damage, increased lipid, protein, and DNA oxidation and oxidative stress, as well as decreased GSH levels. In contrast to FRDA, these pathologies are primarily targeted to the substantia nigra (SN) and also correlate with increased neuronal loss. A hallmark of PD is decreased activity of ETC complex I in the SN of patients. Stack et al. (2008) use a mouse model treated with MPTP, which selectively inhibits the activity of complex I thereby inducing a PD-like phenotype in a mouse model. The inhibition of complex I is associated with increased free radical production, less ATP output, and increases in apoptotic signals cytochrome *c* and caspase-3. Furthermore, the oxidative damage in the SN of PD patients contributes to mtDNA damage as has been seen in FRDA (Bhalla et al., 2016).

These hallmarks of disease pathology in PD mouse SN neurons were suppressed with CSA treatment (Stack et al., 2008). Immunofluorescence detection of mitochondria showed increased mitochondrial number and networking after CSA treatment. Increase in ATP production and decreased levels of 8-OHdG were also quantified. There were reductions also in the levels of cytochrome *c* and caspase-3, indicating greater cell survival. In histological analyses of the PD mouse SN, neurodegeneration was partially alleviated in the CSA treatment group relative to the untreated. CSA treatment further decreased neuronal cell death and preserved projections of the striatal dopaminergic neurons (Stack et al., 2008). The aforementioned use of glutathione metabolism–modulating drugs may be neuroprotective and relieve the pathologies of many CNS diseases.

CSEA/CSA could be a repurposed drug for FRDA as it seems to be neuroprotective for other CNS diseases. Although most antioxidants are for combating free radical stress, supplementing glutathione or targeting its enzymatic cycle also benefits by reversing glutathionylated proteins targeted in disease. Thus, FRDA patients may benefit from targeting glutathione rather than, or in combination with, the iron chelators which are the current methods.

## Concluding Remarks

Iron is central to FRDA pathology in many aspects: (1) iron accumulation in patient brains and hearts implicated in neurologic phenotypes and fibrosis; (2) mitochondrial iron accumulation co-incident with degradation of ATP synthesis and leakage of ROS; and (3) the overall cellular deficiency of ISCs. Furthermore, co-morbid deficiencies in Nrf2 signaling and FXN suppresses the normal antioxidant response needed to deal with the continued oxidative stress (Paupe et al., 2009). However, despite a focus on oxidative stress as a contributor to FRDA, linkage to cytoskeletal abnormalities remains under-appreciated and relatively under investigated. This review was aimed to revisit the under-appreciated findings of ROS stress in disease that strongly impact the cytoskeleton, and to highlight the importance of this aspect of the cell etiology of FRDA in barrier cells, specifically the microvascular endothelial cells of the BBB.

**FIGURE 3:**
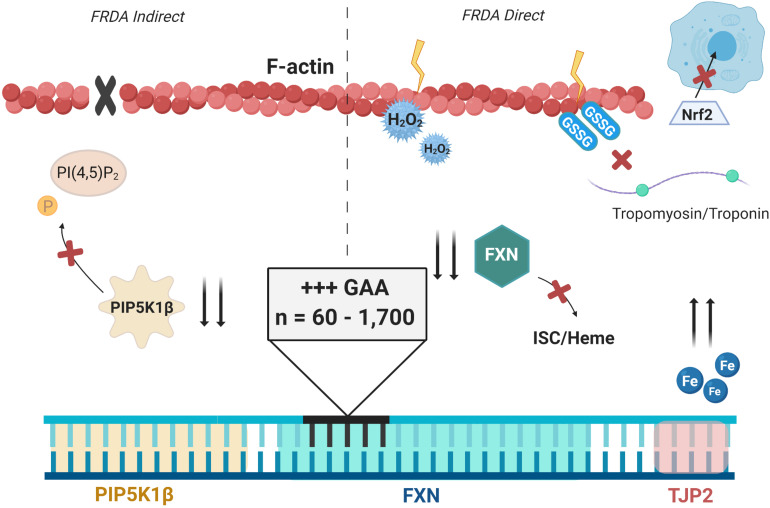
A visual representation of ideas highlighted in this review concerning altered actin physiology in FRDA. The root of these alterations is the increase in GAA expansions in the first intron of FXN, shown in blue. Decreased transcriptional processing of PIP5K1β shown in yellow prevents phosphorylation of PI(4,5)P_2_, preventing normal actin polymerization (left, indirect effect of FRDA). Decreased production of FXN prevents normal ISC and heme biogenesis, permitting free cellular iron. Free radical production and oxidative stress follows, causing direct oxidation of actin filaments and glutathione addition, which prevents polymerization and binding to tropomyosin for stabilization (right, direct effect of FRDA). Nrf2 is shown to have inhibited nuclear translocation. TJP2 also flanks FXN as shown in pink, but is not known to be altered in FRDA at this time.

Oxidative stress in FRDA was first identified by abnormal glutathione presentation in patient blood samples with the ratio being skewed to the oxidized, protein-bound form GSSP rather than its free counterparts GSH or GSSG (Piemonte et al., 2001). Furthermore, morphology of patient fibroblasts was drastically changed due to glutathionylated actin, which could be reversed with the addition of GSH (Pastore et al., 2003). Alterations to actin were mirrored in iron-treated fibroblasts, together confirming that iron-mediated oxidative stress was central to pathophysiology. The indirect downregulation of PIP5K1β in FRDA has also been demonstrated to be pathophysiological due to disrupted signaling of actin dynamics and alterations to cytoskeletal arrangement (Bayot et al., 2013). Furthermore, Nrf2 signaling is hampered by alterations in actin organization, preventing proper mounting of antioxidant responses in FRDA cells (Paupe et al., 2009). The graphical summary presented in Figure 3 illustrates how both direct and indirect pathways lead to the pathological alterations of the actin cytoskeleton in FRDA. The reader is urged to consider the interplay of oxidative stress and cytoskeletal abnormalities that are inherent to the cellular pathophysiology in this disease. Although this topic received some attention nearly two decades ago, it has since been largely neglected. However, in many cell types, particularly barrier cells whose function is fundamentally dependent on the normal modulation of cytoskeletal structure, this aspect of the cellular dysfunction in FRDA may play a particularly significant role in the overall NBIA pathophysiology of this genetic disorder.

## Author Contributions

FS and DK developed the idea for the article. FS performed the majority of the literature search. DK contributed additional topics of interest to be included and funded the acquisition. FS wrote the article with several rounds of editing by DK. Both authors contributed to the article and approved the submitted version.

## Conflict of Interest

The authors declare that the research was conducted in the absence of any commercial or financial relationships that could be construed as a potential conflict of interest.
